# Supplementary material for: Adherence trajectory as an on-treatment risk indicator among drug-resistant TB patients in the Philippines
Source: PLoS One. 2022 Nov 8;17(11):e0277078. doi: 10.1371/journal.pone.0277078 (PMC9642894; doi:10.1371/journal.pone.0277078)
Supplement: S1 File — (DOCX) [file pone.0277078.s001.docx]

Adherence trajectory as an on-treatment risk indicator among drug-resistant TB patients in the Philippines

**SUPPLEMENTAL MATERIAL**

**S T1**, The Philippines Department of Health Treatment Outcome Definitions for DR TB

| Outcome | Definition |
| --- | --- |
| Cure | A patient with bacteriologically confirmed MDR-TB or RR-TB who has completed treatment as recommended by the national policy, without evidence of failure and with three or more consecutive cultures taken at least 30 days apart negative after the intensive phase |
| Treatment completion | Treatment completed as recommended by the national policy without evidence of failure but no record that the three or more consecutive cultures taken at least 30 days apart are negative after the intensive phase. |
| Failed | Any one of the following:  - treatment terminated or need for permanent regimen change;  - lack of evidence of at least two negative cultures (and not followed by a positive culture) by the end of an extended intensive phase (six months) of the shorter regimen; or  - positive sputum smear (confirmed by two consecutive samples) after > 6 months of treatment,  - culture reversion in the continuation phase after conversion to negative;  - evidence of additional acquired resistance to a FQ or a second-line injectable (SLI) drugs; and  - adverse drug reaction resulting to switching to a new regimen. |
| Died | A patient who dies for any reason during the course of treatment |
| Lost to follow-up | A patient whose treatment was interrupted for > 2 consecutive months |
| Not evaluated | A patient for whom no treatment outcomes is assigned. (This includes cases “transferred out” to another treatment unit and whose treatment outcome is unknown.) |

**S T2**, Cross tabulation of binary threshold and trajectory group

|  | 4 weeks | | 8 weeks | | 12 weeks | |
| --- | --- | --- | --- | --- | --- | --- |
|  | AIC | BIC | AIC | BIC | AIC | BIC |
| 2 group model | **9070** | **9133** | **18303** | **18375** | 27561 | 27636 |
| 3 group model | 9116 | 9214 | 18466 | 18576 | **27476** | **27593** |
| 4 group model | No convergence | No convergence | No convergence | No convergence | **27397** | **27555** |
| 5 group model | No convergence | No convergence | No convergence | No convergence | No convergence | No convergence |


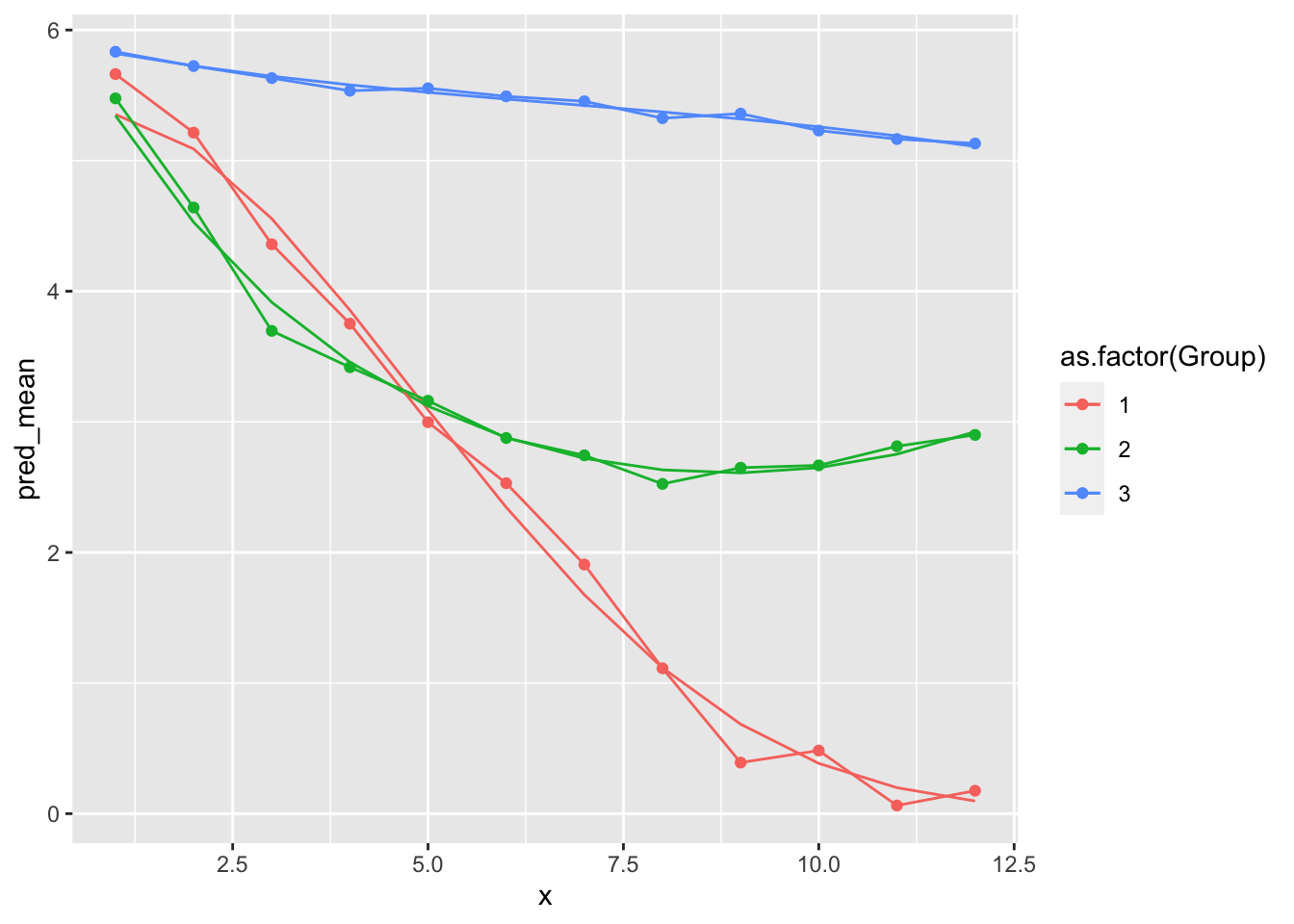


**S F1**, Fit check for 12 week group-based trajectory model. Smooth lines indicate the fitted trend for each trajectory group. Dotted lines indicate the mean value of the raw data at each timepoint within each group.

**S T3**, Coefficients from logistic regression model predicting 6-month treatment outcome based on 12 week adherence pattern, n=596

|  | OR (95% CI) | |
| --- | --- | --- |
|  | Unadjusted | Adjusted |
| High adherence pattern | Reference | Reference |
| Moderate + Low adherence pattern | **3.78 (2.18, 6.53)** | **3.42 (1.90, 6.12)** |

*The adjusted model included patient sex, resistance category (MDR/pre-XDR/XDR), year of treatment initiation, chest x-ray cavitation (no/yes/missing), sputum grade and BMI. This model did not include region due to positivity violations.*
